# Knocking stent release technique to deploy lumen-apposing metal stents during endoscopic ultrasound-guided gallbladder drainage

**DOI:** 10.1055/a-2763-5718

**Published:** 2026-01-08

**Authors:** Takeshi Ogura, Junichi Nakamura, Takafumi Kanadani, Kimi Bessho, Hiroki Nishikawa

**Affiliations:** 1 38588 Pancreatobiliary Advanced Medical Center, Osaka Medical and Pharmaceutical University Hospital, Osaka, Japan; 2 38588 Endoscopy Center, Osaka Medical and Pharmaceutical University Hospital, Osaka, Japan; 3 13010 2nd Department of Internal Medicine, Osaka Medical and Pharmaceutical University, Osaka, Japan

**Fig. 1:**
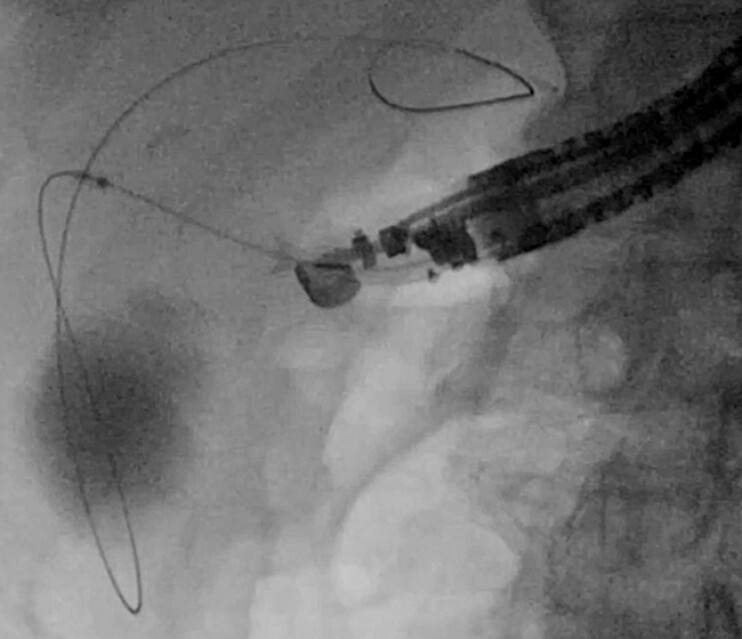
If stent deployment is performed using the scope-pulling technique, stent dislocation may occur as a complication.

Endoscopic ultrasound-guided gallbladder drainage (EUS-GBD) using a lumen-apposing metal stent (LAMS; Hot AXIOS, Boston Scientific, Tokyo, Japan) can be performed in patients with contraindications for surgical resection 1 2 3 4 . EUS-GBD can be performed either from the stomach or the duodenum. Although no randomized trial has compared the optimal drainage route, a recent meta-analysis suggests that the duodenal approach may be safer. Compared with the drainage of walled-off necrosis or a pancreatic pseudocyst, the diameter of the target lesion is usually smaller in EUS-GBD. Therefore, a 10- or 15-mm-diameter LAMS is generally selected. However, compared with a 20-mm-diameter LAMS, the anchoring force may be lower. As a result, if stent deployment is performed using the scope-pulling technique, stent dislocation may occur as a complication ( Fig. 1 ). To overcome this adverse event, we usually deploy the LAMS using the “knocking stent release technique.” Technical tips for the knocking stent release technique are presented.

**Fig. 2:**
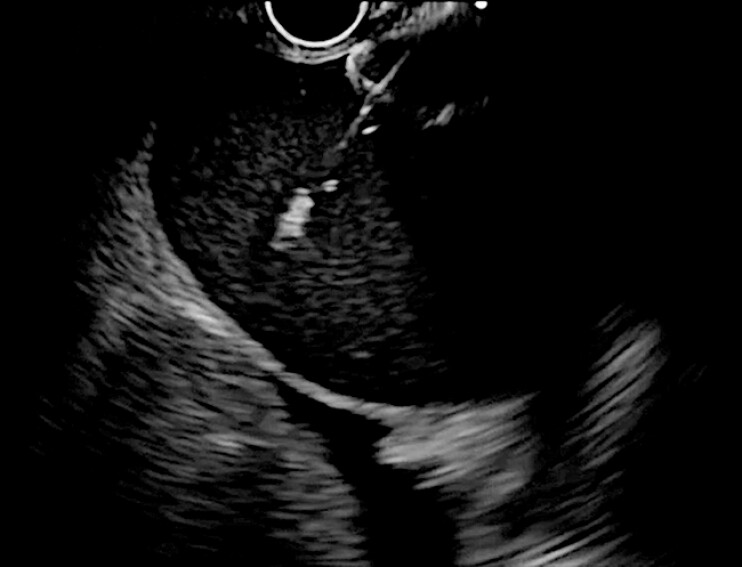
The proximal flange is released using the intra-scope channel release technique.

**Fig. 3:**
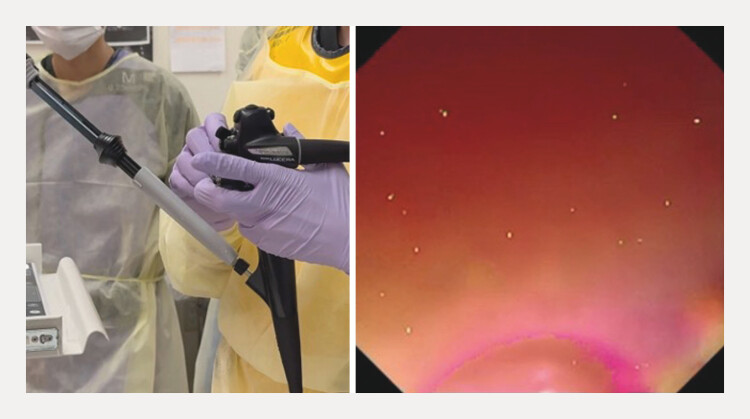
The right angle of the echoendoscope is applied.

**Fig. 4:**
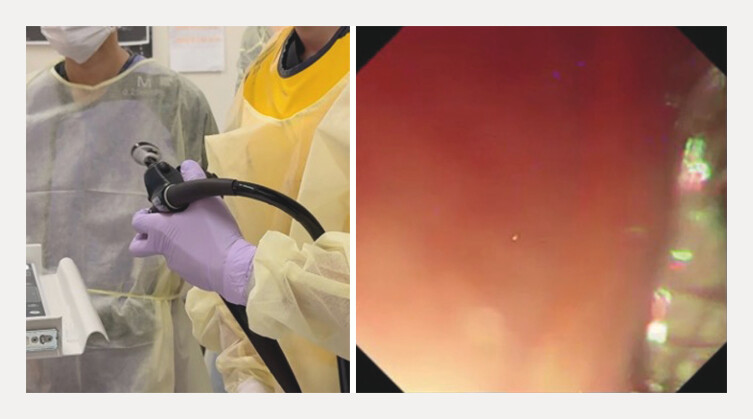
The scope is rotated clockwise.

**Fig. 5:**
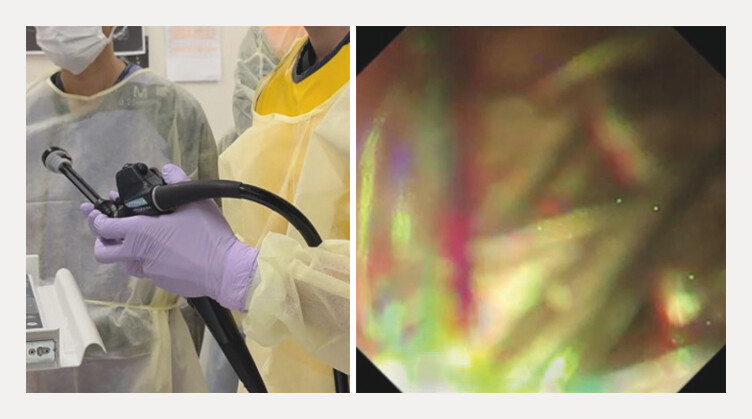
The stent delivery system is knocking, and the stent is extracted from the echoendoscope.

An echoendoscope was inserted into the duodenum, and the gallbladder was identified. To prevent double mucosal penetration, the delivery system was advanced to the duodenal wall using the water-filling technique. Then, the delivery system insertion with electrocautery dilation was performed, with successful insertion into the gallbladder. Subsequently, the distal flange was released, and the delivery system was pulled back to the proximal gallbladder wall. Finally, the proximal flange was released using the intra-scope channel release technique ( Fig. 2 ). Next, if the stent release was performed by pulling the echoendoscope, stent dislocation can occur; therefore, first, the right angle of the echoendoscope was applied ( Fig. 3 ), and the scope was then rotated clockwise ( Fig. 4 ). By doing so, the LAMS was partially visualized. Finally, the stent delivery system was knocking, and the stent was finally extracted from the echoendoscope without any adverse events ( Fig. 5 ; Video 1 ).

The stent delivery system is knocking, and the stent is extracted from the echoendoscope.Video 1

In conclusion, the knocking stent release technique to deploy a LAMS during EUS-GBD might be useful.

Endoscopy_UCTN_Code_TTT_1AS_2AH
